# A novel 10 glycolysis-related genes signature could predict overall survival for clear cell renal cell carcinoma

**DOI:** 10.1186/s12885-021-08111-0

**Published:** 2021-04-09

**Authors:** Qianwei Xing, Tengyue Zeng, Shouyong Liu, Hong Cheng, Limin Ma, Yi Wang

**Affiliations:** 1 Department of Urology, Affiliated Hospital of Nantong University, No.20 West Temple Road, Nantong, Jiangsu Province, 226001 China; 2 Department of Urology, The First Affiliated Hospital of Nanjing Medical University, Nanjing, Jiangsu Province, 210029 China; 3 Department of Urology, Zhongda Hospital Affiliated to Southeast University, Nanjing, 210009 China

**Keywords:** Glycolysis, Overall survival, Prognosis, Signature, Clear cell renal cell carcinoma

## Abstract

**Background:**

The role of glycolysis in tumorigenesis has received increasing attention and multiple glycolysis-related genes (GRGs) have been proven to be associated with tumor metastasis. Hence, we aimed to construct a prognostic signature based on GRGs for clear cell renal cell carcinoma (ccRCC) and to explore its relationships with immune infiltration.

**Methods:**

Clinical information and RNA-sequencing data of ccRCC were obtained from The Cancer Genome Atlas (TCGA) and ArrayExpress datasets. Key GRGs were finally selected through univariate COX, LASSO and multivariate COX regression analyses. External and internal verifications were further carried out to verify our established signature.

**Results:**

Finally, 10 GRGs including ANKZF1, CD44, CHST6, HS6ST2, IDUA, KIF20A, NDST3, PLOD2, VCAN, FBP1 were selected out and utilized to establish a novel signature. Compared with the low-risk group, ccRCC patients in high-risk groups showed a lower overall survival (OS) rate (*P* = 5.548Ee-13) and its AUCs based on our established signature were all above 0.70. Univariate/multivariate Cox regression analyses further proved that this signature could serve as an independent prognostic factor (all *P* < 0.05). Moreover, prognostic nomograms were also created to find out the associations between the established signature, clinical factors and OS for ccRCC in both the TCGA and ArrayExpress cohorts. All results remained consistent after external and internal verification. Besides, nine out of 21 tumor-infiltrating immune cells (TIICs) were highly related to high- and low- risk ccRCC patients stratified by our established signature.

**Conclusions:**

A novel signature based on 10 prognostic GRGs was successfully established and verified externally and internally for predicting OS of ccRCC, helping clinicians better and more intuitively predict patients’ survival.

**Supplementary Information:**

The online version contains supplementary material available at 10.1186/s12885-021-08111-0.

## Background

Renal cell carcinoma (RCC) incidence is second only to bladder cancer and there will be 73,750 newly estimated cases and 14,830 estimated death in the United States, 2020 [1]. Therein, clear cell RCC (ccRCC) accounts for approximately 70–80% of the pathological subtype of RCC. Due to the insensitivity of radiotherapy or chemotherapy in RCC, its therapy is mainly surgical treatment. At present, survival rates of metastatic kidney cancer are still low. Its two-year survival is lower than 20%, and about one-third of patients are found to have tumor metastasis when diagnosed [2, 3]. Therefore, further understanding of their molecular mechanisms and development of effective early screening and diagnosis methods are essential for improving the treatment effect and life quality for these patients.

In recent years, there have been more and more researches on the metabolic changes of tumor cells. Warburg effect is the most common and widely studied metabolic change in cancer cells, that is, in the presence of oxygen, malignant tumor cells have an inherent tendency to incompletely oxidize glucose [4]. Studies have revealed that many tumors have enhanced glucose uptake in adjacent tissues, and high glucose uptake rates are simultaneously present with suppressed glucose oxidation [4]. Glucose is converted into lactic acid during glycolysis, and cancer cells obtain maximum energy in this way. This phenomenon is ubiquitous in tumors [5], and this metabolic change is particularly noticeable in kidney cancer [6].

Accumulating evidence have reported that glycolysis-related genes (GRGs) are differently expressed in various malignant cancers and play important roles in tumorigenesis and progress. For example, studies have presented that about 90% of patients with sporadic ccRCC have mutations of VHL gene [7]. When this gene is deleted, HIF-α accumulates, and genes such as VEGF, PDGF, TGF-α, and MMP are activated to participate in neovascularization formation, cell proliferation, infiltration and distant metastasis promote the development of tumors [8]. One of the key enzymes of glycolysis, hexokinase 2 (HK2), has turned out to be abnormally expressed in ccRCC and can promote cell proliferation and invasion [9]. Multiple genes, such as FBP1, PLOD2, VCAN, and CD44 have been demonstrated to participate in epithelial-mesenchymal transition (EMT) promotion of tumor metastasis [10–13]. More and more researches on the relationships between glycolysis and tumor occurrence and development have emerged. Glycolysis roles in the progress of RCC have also been confirmed. However, there is currently no prognostic model for ccRCC based on GRGs. Hence, this article is committed to establish a novel GRGs-related prognostic signature and to explore its associations with immune infiltration for predicting ccRCC patients’ overall survival (OS).

## Methods

### Data collection and identification of differentially expressed GRGs

Clinical information and RNA-sequencing FPKM values of 539 ccRCC tumor samples and 72 normal tissues were obtained from The Cancer Genome Atlas (TCGA, https://portal.gdc.cancer.gov/) dataset, with a mixture of different histologic types of ccRCC included in this study. All raw data were pre-processed by normalization, log 2 transformation and excluding average count values of genes < 1. Differentially expressed GRGs were identified by the “Limma” package, in setting the cut-off values of |log2 fold change |>1 and false discovery rate (FDR) < 0.05. In addition, the E-MTAB-1980 dataset from the ArrayExpress database (https://www.ebi.ac.uk/arrayexpress/) including 99 ccRCC tumor samples were served as an external verification cohort.

### Gene ontology (GO) and Kyoto encyclopedia of genes and genome (KEGG) pathway enrichment analyses

Through the enrichment of GO and KEGG pathway enrichment analyses, differently expressed GRGs’ biological functions were comprehensively evaluated. Therein, GO analysis terms included molecular functions (MF), cellular components (CC) and biological processes (BP). All functional annotation and pathway enrichment analyses were conducted using the R package of “clusterProfiler”.

### Protein-protein interaction (PPI) network and related module screening

In order to evaluate the PPI network, we submitted these differentially expressed GRGs to the online tool STRING (http://www.string-db.org/) and visualized the PPI network by Cytoscape 3.7.0 software. By means of the Molecular Complex Detection (MCODE) plug-in, we screened out the top three hub modules identified from the PPI network with the cut-off criteria of a node degree > 3, a combined score > 0.9 and *P* < 0.05.

### Prognostic model construction and validation

We utilized data in the TCGA database as the training queue and the data in the ArrayExpress database as the external verification queue. Univariate Cox regression was performed to analyze associations between differently expressed GRGs and OS for ccRCC in the TCGA database, and 40 candidate genes were selected. The least absolute contraction and selection operator (LASSO) method was used to improve accuracy and to select out the optimal gene combination. Finally, 10 genes were screened out by multivariate Cox regression analysis to establish a signature. According to the genes’ expression levels and their regression coefficients, a risk signature is established. The riskscore formula for each ccRCC patient was displayed as following:

$$ Riskscore={\sum}_{i= 1}^n expi\ast \beta i. $$

Therein, β represented the regression coefficient and exp. was the genes’ expression levels. Taking the median riskScore in the TCGA training database as the threshold, the eligible ccRCC patients were classified into a high-risk and a low-risk group, and Kaplan-Meier survival curve was drawn to show the prognosis difference between the two groups of patients. Besides, our established signature was carefully evaluated in three validations sets including the external validation dataset (ArrayExpress), the internal validation dataset 1 (test 1) as well as the internal validation dataset 2 (test 2).

### Univariate/multivariate cox regression analyses and nomogram construction

Univariate and multivariate Cox regression analyses were applied to evaluate whether our established signature and various clinical parameters could be served as an independent prognostic factor for ccRCC. Six clinical factors and riskscore were utilized to establish the prognostic nomogram, evaluating the ccRCC patients’ 1-, 3- and 5-year OS. C-index and the area under the curve (AUC) were calculated to assess nomogram accuracy. Calibration charts were applied to intuitively assess nomogram prognostic ability. In addition, the prognosis nomogram was also verified in external validation cohort from ArrayExpress database.

### Verification of the GRGs’ protein expression in HPA database and survival analysis in Kaplan-Meier plotter website

By means of the Human Protein Atlas (HPA) dataset (http://www.proteinatlas.org/), we validated the 10 hub GRGs’ protein expression levels in ccRCC by immunohistochemical staining. Kaplan–Meier plotter website (http://kmplot.com/analysis/) was also applied to assess the prognosis of these 10 hub GRGs in ccRCC by survival analysis.

### Estimation of tumor-infiltrating immune cells (TIICs)

The TIICs expression levels in all ccRCC samples were calculated by the “limma” package of R software. Then, algorithm with LM22 gene signature and 1000 permutations were calculated to find whether these TIICs are highly related to high- and low- groups stratified by our established signature [14]. *P*-value below 0.05 above mentioned was set as threshold.

### Statistical analysis

R software 3.6.3 was utilized to calculate all statistical analyses. Survival curves were drawn by the Kaplan-Meier method with log-rank test. Univariate COX, LASSO and multivariate COX regression analyses were utilized to calculate the regression coefficient and to establish a signature. All statistical tests are bilateral. P-value below 0.05 was regarded to be statistically significant.

## Results

### Identification of differently expressed GRGs

**Fig. 1:**
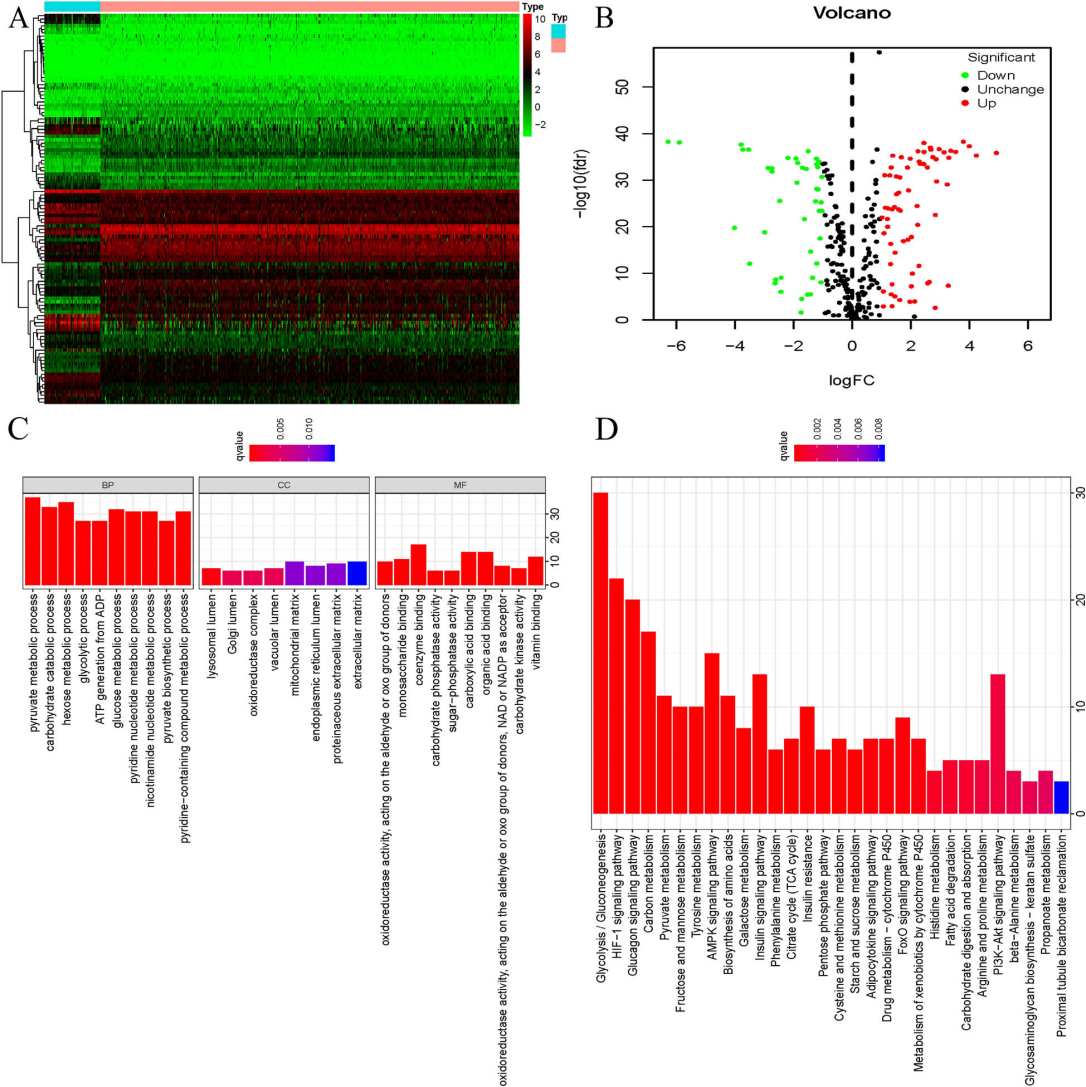
The differentially expressed GRGs identified in ccRCC. **a** Heatmap; **b** Volcano plot. The red nodes represent the up-regulated genes Green nodes represent the down-regulated genes (*P* value < 0.05 and |log2(FC)| > 1); **c** GO enrichment of differently expressed GRGs; **d** KEGG pathway analysis of differently expressed GRGs

The workflow of our study was presented in Supplement Figure S1. A total of 611 patients were extracted from the TCGA database, containing 539 renal cancer samples and 72 normal specimens with a mixture of different histologic types of ccRCC included in this study. The R package of “Limma” was applied to discover differentially expressed mRNAs (DE-mRNAs) with a thresholds of |log 2 (FC)| > 1 as well as FDR less than 0.05, 113 differently expressed GRGs were screened from the GRG list, including 44 down-regulated and 69 up-regulated GRGs. Their expression heatmap and volcano plot were shown in Fig. 1a-b.

### Functional annotation and pathway enrichment

Results of GO and KEGG analyses presented that differently expressed GRGs were remarkably enriched in the biological processes (BP) analysis associated with carbohydrate catabolic process, pyruvate metabolic process, glycolytic process, hexose metabolic process, ATP generation from ADP, pyridine nucleotide metabolic process, glucose metabolic process, pyridine−containing compound metabolic process, nicotinamide nucleotide metabolic process pyruvate biosynthetic process. Through the molecular function (MF) analysis, they were significantly enriched in oxidoreductase activity, monosaccharide binding, coenzyme binding, carbohydrate phosphatase activity, organic acid binding, sugar−phosphatase activity carboxylic acid binding, oxidoreductase activity, NAD or NADP as acceptor, carbohydrate kinase activity, vitamin binding. Concerning the cellular component (CC), differently expressed GRGs were notably enriched in lysosomal lumen, Golgi lumen, oxidoreductase complex, vacuolar lumen, mitochondrial matrix, proteinaceous extracellular matrix, endoplasmic reticulum lumen, extracellular matrix. KEGG pathway analysis is significantly enriched in HIF-1 signaling pathway, Glycolysis/Gluconeogenesis, Carbon metabolism, Glucagon signaling pathway, Pyruvate metabolism, Fructose and mannose metabolism, Tyrosine metabolism, AMPK signaling pathway, Biosynthesis of amino acids, Galactose metabolism, Insulin signaling pathway, suggesting that these prognostic genes are indeed associated with glycolysis (Fig. 1c-d).

### PPI network integration and the top 3 key modules selection

**Fig. 2:**
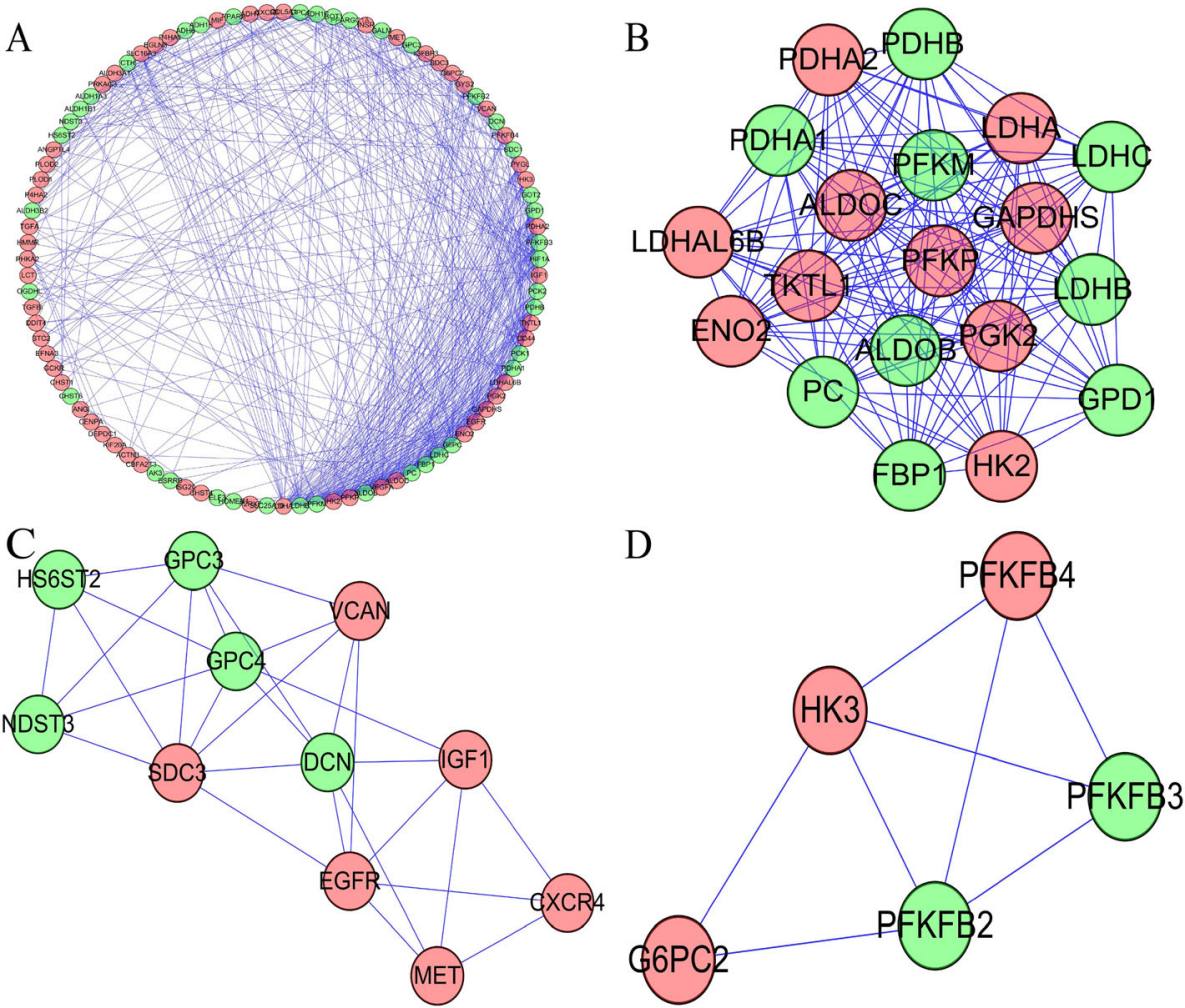
Protein–protein interaction network and its top 3 key modules. **a** Protein–protein interaction network of differentially expressed GRGs. **b**-**c** The top three key modules from PPI network

In order to further study their roles of differentially expressed GRGs in RCC, we used the STRING database to reveal the relationships between differentially expressed GRGs and visualized by Cytoscape software (Fig. 2a). We also used Cytoscape’s MODE tool to process the co-expression network and to identify the top three key modules in the PPI network (Fig. 2b-d).

### Construction a prognostic model

**Fig. 3:**
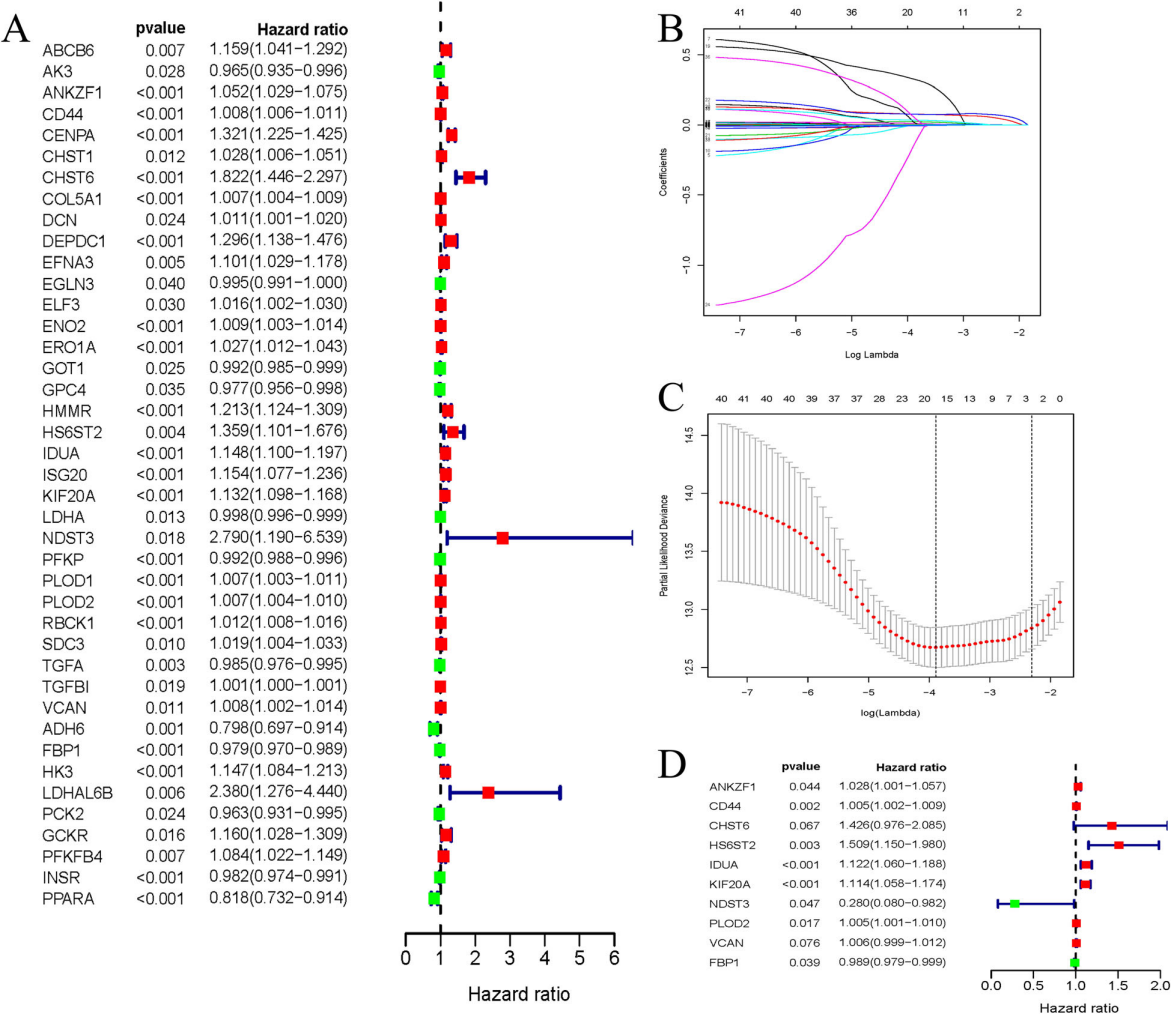
Construction a prognostic signature using univariate Cox regression analysis, LASSO analysis and multivariate Cox regression analysis. **a** Risk ratio forest plot showed the prognostic value of 40 candidate genes screened out by univariate Cox regression. **b**-**c** LASSO coefficients profiles of 20 GRGs; The partial likelihood deviance plot displayed the minimum number corresponds to the covariates utilized for multivariate Cox analysis. **d** Risk ratio forest plot showed the prognostic value of 10 prognostic genes screened out by multivariate Cox regression

To construct a prognostic model, univariate regression analysis was done to find out 40 candidate GRGs (Fig. 3a). The LASSO Cox regression model was applied to avoid overfitting of the model (Fig. 3b-c). Finally, 10 genes were screened out by multivariate Cox regression analysis to establish a signature (Fig. 3d and Table 1). The riskscores of each patient were shown as following:

$$ \mathrm{Riskscore}=\left(0.0280\times \mathrm{Exp}\ \mathrm{ANKZF}1\right)+\left(0.0054\times \mathrm{Exp}\ \mathrm{CD}44\right)+\left(0.3550\times \mathrm{Exp}\ \mathrm{CHST}6\right)+\left(0.4116\times \mathrm{Exp}\ \mathrm{HS}6\mathrm{ST}2\right)+\left(0.1155\times \mathrm{Exp}\ \mathrm{IDUA}\right)+\left(0.1080\times \mathrm{Exp}\ \mathrm{KIF}20\mathrm{A}\right)+\left(-1.274\times \mathrm{Exp}\ \mathrm{NDST}3\right)+\left(0.0052\times \mathrm{Exp}\ \mathrm{PLOD}2\right)+\left(0.0059\times \mathrm{Exp}\ \mathrm{VCAN}\right)+\left(-0.011\times \mathrm{Exp}\ \mathrm{FBP}1\right). $$

**Table 1:** Coefficients and HR of the 10 key prognostic GRGs

gene	coef	HR	HR.95 L	HR.95H	p-value
ANKZF1	0.02801704	1.02841321	1.0008025	1.05678565	0.0436188
CD44	0.00539614	1.00541073	1.00198434	1.00884883	0.00194757
CHST6	0.35495268	1.42611317	0.97559469	2.08467593	0.06689023
HS6ST2	0.4115756	1.50919379	1.15040925	1.97987446	0.0029622
IDUA	0.11551555	1.12245197	1.06029044	1.18825784	7.07E-05
KIF20A	0.10802281	1.11407316	1.05755908	1.17360724	4.76E-05
NDST3	−1.2741372	0.27967216	0.07967676	0.98167295	0.04671924
PLOD2	0.00521398	1.0052276	1.0009296	1.00954405	0.01707989
VCAN	0.00589737	1.0059148	0.99937923	1.0124931	0.07618717
FBP1	− 0.0106882	0.98936868	0.97935727	0.99948242	0.03942413

### Validation the expression and prognosis of key GRGs

**Fig. 4:**
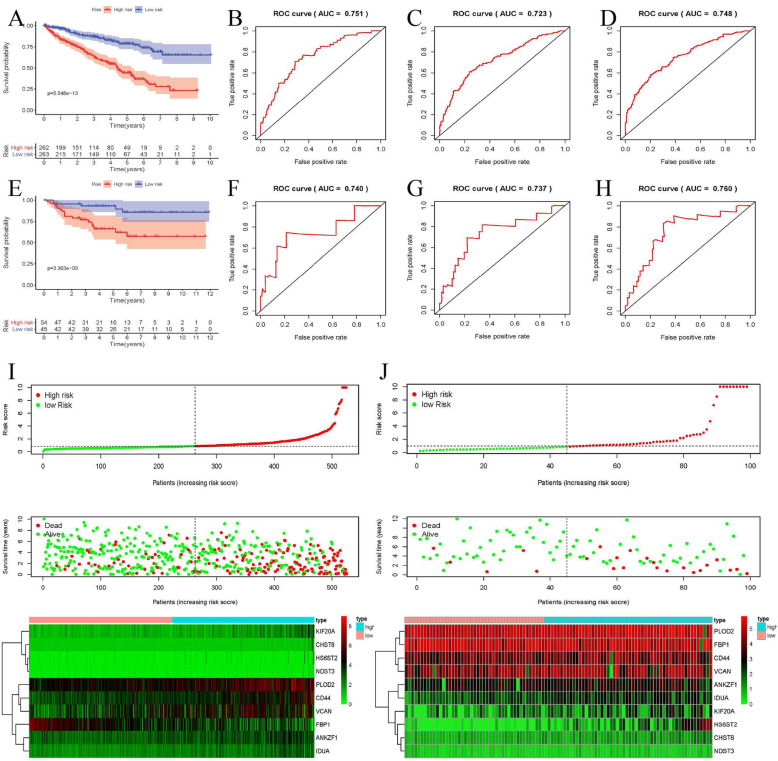
Evaluation and external verification of six RBPs established signature; **a** Kaplan-Meier survival curves for low- and high-risk subgroups stratified by riskscore signature in the training dataset (TCGA). **b**-**d** ROC curves for forecasting 1-year, 3-year and 5-year OS based on risk score in the TCGA training dataset. **e** Kaplan-Meier survival curves for low- and high-risk subgroups stratified by riskscore signature in the external validation dataset (ArrayExpress); **f**-**h** ROC curves for forecasting 1-year, 3-year and 5-year OS based on risk score in the external validation dataset (ArrayExpress); **i**-**j** Expression heat map, risk score distribution, and survival status in the training dataset (TCGA) and the external validation dataset (ArrayExpress)

**Fig. 5:**
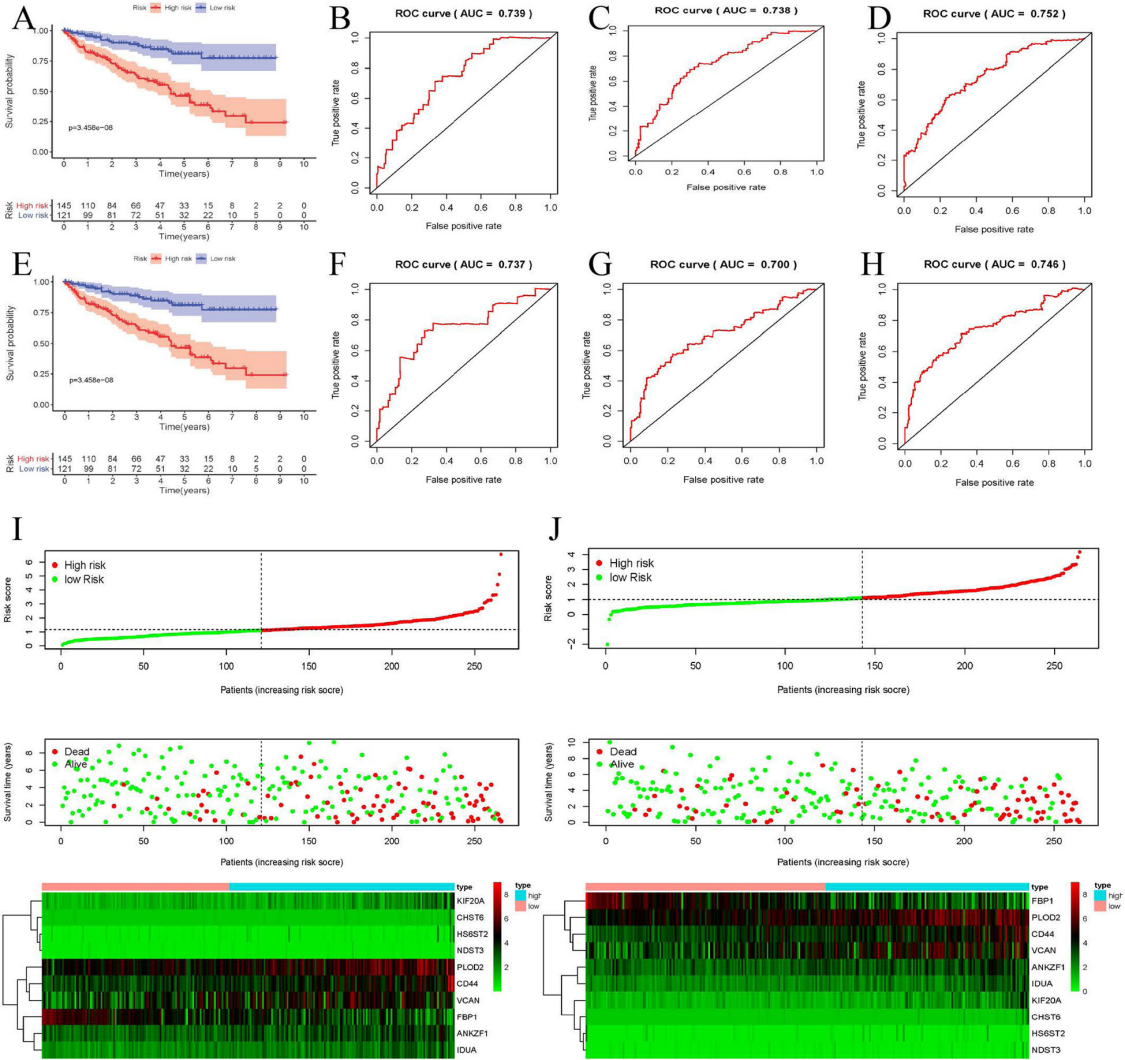
Internal verification of 10 GRGs based riskscore signature. **a** Kaplan-Meier survival curves for low- and high-risk subgroups stratified by riskscore signature in the internal validation dataset 1; **b**-**d** ROC curves for forecasting 1-year, 3-year and 5-year OS based on risk score in the internal validation dataset 1; **e** Kaplan-Meier survival curves for low- and high-risk subgroups stratified by riskscore signature in the internal validation dataset 2; **f**-**h** ROC curves for forecasting 1-year, 3-year and 5-year OS based on risk score in the internal validation dataset 2; **i**-**j** Expression heat map, risk score distribution, and survival status in the internal validation dataset 1 and in the internal validation dataset 2

Based on our established glycolysis signature, 539 ccRCC cases were subdivided into a high-risk and a low-risk group, accordingly to the median riskscore. Kaplan-Meier survival analysis indicated that ccRCC patients in low-risk groups could have a markedly longer OS than patients in high-risk groups (*P* = 5.548e− 13, Fig. 4a). The signature showed superior predictive veracity of patients’ OS with the 1-, 3- and 5-year AUC values of 0.724, 0.716 and 0.741, separately (Fig. 4b-d, Table 2). Besides, the riskscore for each sample was also calculated and ranked and the heatmap showed the expression value of ten significant GRGs between high- and low-risk groups. As the risk score increased, ccRCC patients would have a shorter survival time and more dead events (Fig. 4i). Three validation datasets containing the external validation dataset, the internal validation dataset 1 as well as the internal validation dataset 2 were used to verify the risk score signature had similar predictive values in different populations. Survival analysis showed that all three testing cohorts had similar outcomes (Fig. 4e Fig. 5a and e). The 1-, 3- and 5-year AUC values of OS in three testing cohorts were all above 0.70, also showed a favorable predictive ability (Figs. 4f-h, Fig. 5, Table 2). The expression heatmap of ten hub GRGs, survival status and risk score of signature of ccRCC patients were displayed in Fig. 5.

**Table 2:** External and internal verification datasets of 1-year, 3-year, 5-year ROC

Dataset	1-year ROC	3-year ROC	5-year ROC
The whole training dataset (TCGA)	0.751	0.723	0.748
The external validation dataset (ArrayExpress)	0.740	0.737	0.760
The internal validation dataset 1 (test 1)	0.739	0.738	0.752
The internal validation dataset 2 (test 2)	0.737	0.700	0.746

### Determination of the GRGs risk model as an independent prognostic factor

**Fig. 6:**
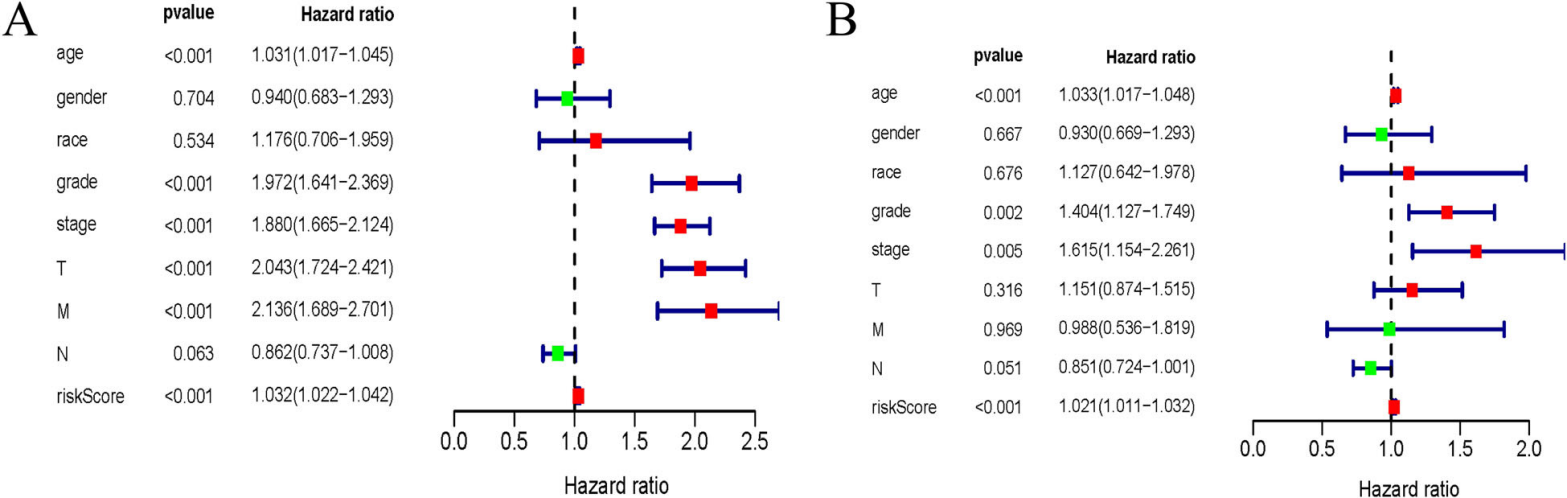
Independent prognostic factor evaluation. **a** Univariate cox regression analysis of the training dataset (TCGA). **b** Multivariate cox regression analysis of the training dataset (TCGA)

Univariate and multivariate Cox regression analyses were applied to evaluate whether our established signature and various clinical parameters could be served as an independent prognostic factor for ccRCC. In the TCGA cohort, univariate analysis showed the glycolysis signature, age, grade, stage, T and M were all remarkably related to OS (all *P* < 0.001). Multivariate analysis indicated that the glycolysis signature, age, grade and stage were all marked correlated with OS (all *P* < 0.01). Therefore, the prognostic glycolysis signature constructed by the TCGA training set was an independent prognostic factor for ccRCC (Fig. 6, Table 3).

**Table 3:** Univariate and multivariate Cox regression analysis of external and internal verification datasets for overall survival (OS) in TCGA training dataset

id	Univariate Cox regression analysis				Multivariate Cox regression analysis			
	HR	HR.95 L	HR.95H	pvalue	HR	HR.95 L	HR.95H	*p* value
age	1.030913	1.017301	1.044707	**7.14E-06**	1.032813	1.01749	**1.048366**	**2.30E-05**
gender	0.939895	0.682968	1.293475	0.703594	0.9303	0.669431	1.292828	0.666978
race	1.175718	0.705799	1.958508	0.534112	1.127239	0.642321	1.978247	0.676404
grade	1.971561	1.640971	2.368751	**4.20E-13**	1.403862	1.126968	1.74879	**0.002475**
stage	1.880446	1.664529	2.12437	**3.38E-24**	1.615246	1.154106	2.260642	**0.00518**
T	2.043146	1.72421	2.421079	**1.57E-16**	1.150896	0.874324	1.514955	0.316239
M	2.135679	1.688608	2.701115	**2.42E-10**	0.987786	0.536484	1.81873	0.968524
N	0.862088	0.737405	1.007852	0.062627	0.85109	0.723966	1.000537	0.050765
riskScore	1.031719	1.021717	1.041819	**3.33E-10**	1.021333	1.010815	1.03196	**6.42E-05**

### Nomogram construction based on the clinical characteristic and ten GRGs’ signature

**Fig. 7:**
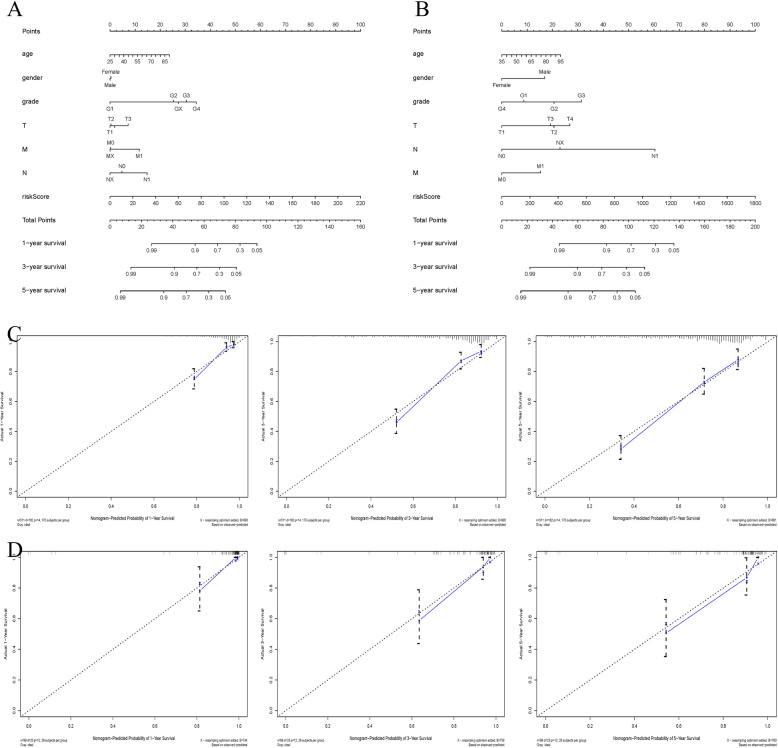
Nomogram to predict the 1-, 3-, and 5-year OS of ccRCC patients in TCGA and ArrayExpress databases; **a**-**b** Nomogram for predicting probabilities of patients with ccRCC with 1-, 3-, 5-year OS in the TCGA and ArrayExpress databases respectively; **c** Calibration plot of the nomogram for agreement test between 1-,3- and 5-year OS prediction and actual outcome in the TCGA training cohort. **d** Calibration plot of the nomogram for agreement test between 1-,3- and 5-year OS prediction and actual outcome in external validation dataset (ArrayExpress)

To predict ccRCC patients’ prognosis, a prognostic nomogram was constructed by TCGA dataset to predict the 1-, 3- and 5-year OS for ccRCC. Severn prognostic parameters were included in the prediction model, including the riskScore, age, gender, grade, T, M and N (Fig. 7a). The nomogram showed good predictive power of 1-, 3- and 5-year OS rates for ccRCC. The 1-, 3- and 5-year AUC and their C-index were 0.836, 0.799, 0.765 and 0.781 in the TCGAs database respectively (Table 4, Supplement Figure S2). Calibration charts (Fig. 7c) showed that the 1-, 3- and 5-year survival rates of the TCGA cohort predicted were in excellently consistent with actual observations. In addition, we constructed another prognosis nomogram as an external verification set to verify the previous results in ArrayExpress dataset (Fig. 7b). Its C-index was 0.86 and the AUC values were 0.888, 0.915, and 0.902 (Table 4, Supplement Figure S2) respectively, indicating that the nomogram also has good predictive power for OS rates in the testing set. The calibration chart shows excellent agreement between the 1-, 3- and 5-year OS predictions and actual observations of ccRCC patients in the external testing set (Fig. 7d).

**Table 4:** 1-year, 3-year, 5-year ROC and C-index of nomogram for in the training dataset (TCGA) and the external validation dataset (ArrayExpress)

	1-year ROC	3-year ROC	5-year ROC	C-index
TCGA cohort	0.836	0.799	0.765	0.781
ArrayExpress cohort	0.888	0.915	0.902	0.868

### Prognostic value of 10 key GRGs and their associations between our established signature and clinical factors

**Fig. 8:**
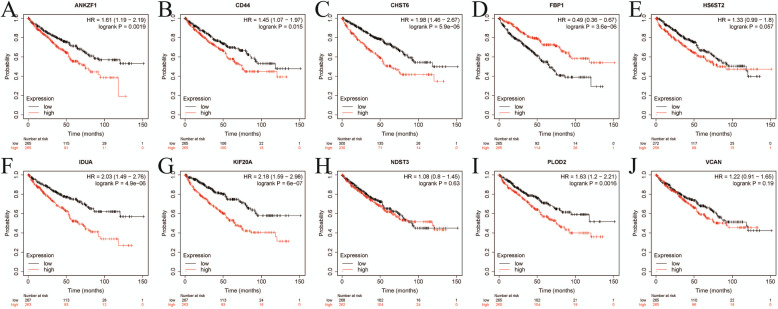
Survival analyses of 10 GRGs by Kaplan-Meier Plotter website; **a** ANKZF1; **b** CD44; **c** CHST6; **d** FBP1; **e** HS6ST2; **f** IDUA; **g** KIF20A; **h** NDST3; **i** PLOD2; **j** VCAN

Based on the median expression, survival analyses of 10 GRGs (ANKZF1, CD44, CHST6, HS6ST2, IDUA, KIF20A, NDST3, PLOD2, VCAN, FBP1) by Kaplan-Meier Plotter website were displayed in Fig. 8. The correlations between our established and clinical factors were also analyzed and revealed that the established signature was firmly associated with grade (*P* = 0.030), stage (*P* = 0.036) and staged T (*P* = 0.042) (Table 5, Supplementary Figure S3).

**Table 5:** Clinical correlation analysis between 10 prognostic GRGs, riskscore and clinical features

id	age	gender	race	grade	stage	T	M	N
**ANKZF1**	−0.511 (0.610)	0.375 (0.708)	5.65 (0.059)	**−2.764 (0.006)**	**−3.114 (0.002)**	**−2.766 (0.006)**	**−3.05 (0.003)**	−0.075 (0.940)
**CD44**	−0.342 (0.733)	**−2.495 (0.013)**	4.916 (0.086)	**−2.216 (0.027)**	**− 2.172 (0.031)**	**− 2.268 (0.024)**	−1.832 (0.070)	0.795 (0.427)
**CHST6**	−1.198 (0.233)	−0.024 (0.981)	5.346 (0.069)	−1.743 (0.082)	−1.794 (0.074)	−1.872 (0.063)	0.193 (0.847)	0.581 (0.561)
**HS6ST2**	−0.236 (0.814)	1.4 (0.163)	1.036 (0.596)	0.206 (0.837)	−0.313 (0.755)	−0.593 (0.554)	− 0.12 (0.905)	−0.394 (0.694)
**IDUA**	−0.556 (0.579)	1.105 (0.270)	**13.523 (0.001)**	**−4.243 (2.673e-05)**	**−4.079 (5.649e-05)**	**−3.349 (9.142e-04)**	**−3.78 (2.389e-04)**	−0.132 (0.895)
**KIF20A**	−0.84 (0.402)	**−2.636 (0.009)**	**16.433 (2.701e-04)**	**−5.354 (1.566e-07)**	**−4.874 (1.993e-06)**	**− 4.542 (9.366e-06)**	**− 2.609 (0.010)**	− 0.112 (0.911)
**NDST3**	−0.454 (0.650)	− 0.447 (0.655)	0.183 (0.912)	**−2.118 (0.035)**	−1.423 (0.156)	− 1.434 (0.153)	− 1.32 (0.189)	**2.511 (0.013)**
**PLOD2**	0.171 (0.865)	**−2.622 (0.009)**	**9.262 (0.010)**	**−2.86 (0.004)**	**−4.439 (1.319e-05)**	**−4.286 (2.663e-05)**	**−2.177 (0.031)**	1.353 (0.177)
**VCAN**	−0.054 (0.957)	**−2.013 (0.045)**	5.449 (0.066)	−1.422 (0.156)	−1.507 (0.133)	− 1.453 (0.147)	−0.316 (0.752)	0.947 (0.344)
**FBP1**	1.325 (0.186)	**4.761 (3.75e-06)**	5.32 (0.070)	**3.295 (0.001)**	**3.412 (7.026e-04)**	**3.303 (0.001)**	0.967 (0.335)	0.976 (0.330)
**riskScore**	−1.338 (0.183)	−1.399 (0.163)	0.705 (0.703)	**−2.178 (0.030)**	**− 2.111 (0.036)**	**− 2.048 (0.042)**	−0.851 (0.396)	−0.655 (0.513)

### Validation of the expression of 10 critical GRGs in ccRCC from HPA database

As detailed in Supplementary Figure S4, immunohistochemical staining of these 10 critical GRGs from HPA database were utilized to verify their protein expressions. Compared with normal kidney tissues, antibody stainings for ANKZF1, CD44, IDUA, KIF20A, PLOD2, and VCAN were high in ccRCC tumor tissues, whereas they were low for CHST6, HS6ST2, NDST3 and FBP1.

### Clinical factors stratified by the riskScore for OS

**Fig. 9:**
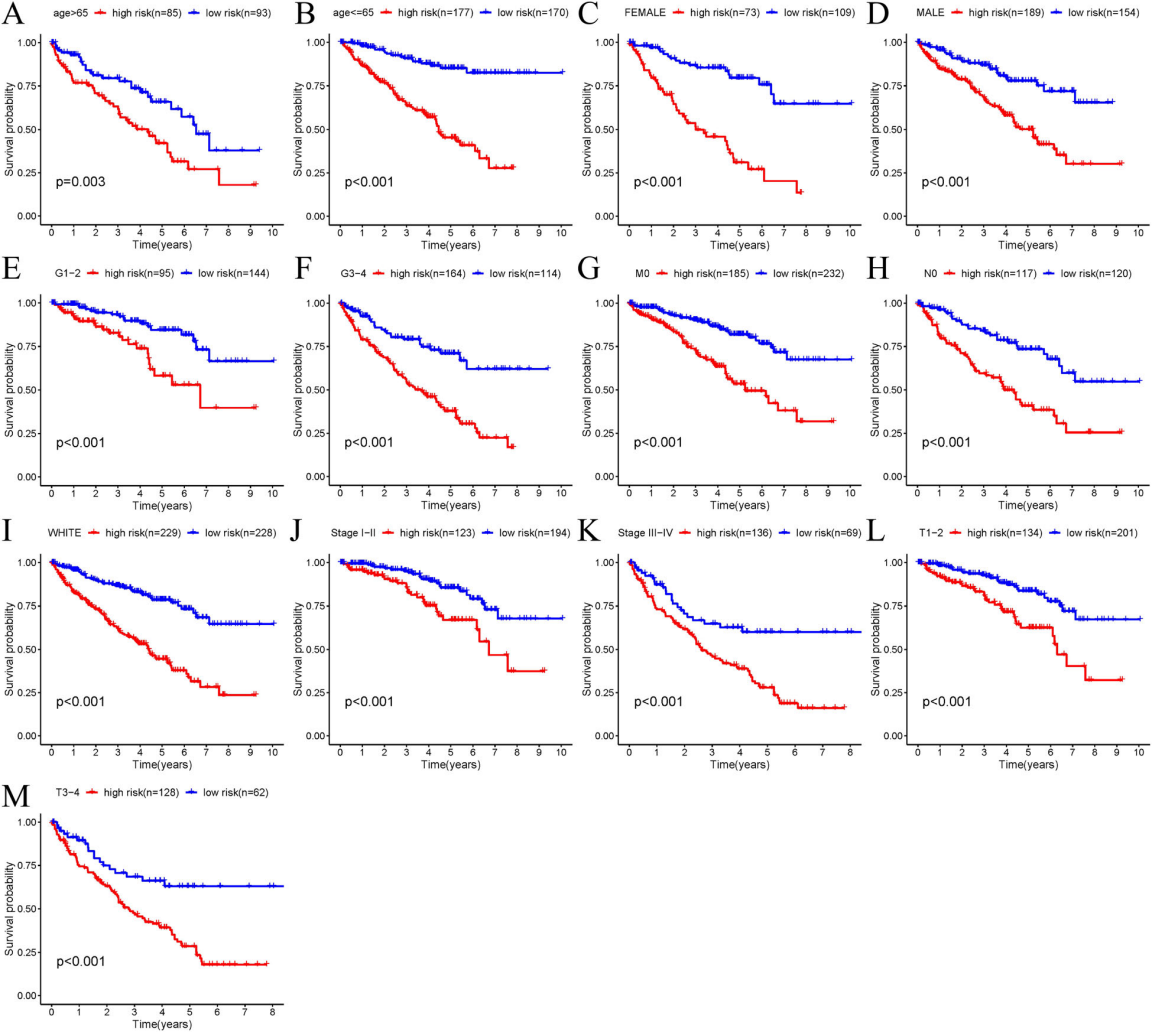
Clinicopathological parameters stratified by our established riskScore for OS; **a** age > 65; **b** age ≤ 65; **c** Female; **d** Male; **e** Grade1–2; **f** Grade3–4; **g** M0; **h** N0; **i** White; **j** Stage I-II; **k** Stage III-IV; **l** T1–2 stage; **m** T3–4 stage

Our results indicated that our established riskScore was capable of predicting OS in age ≤ 65, age > 65, Female, Male, Grade 1–2, Grade 3–4, M0, N0, White, Stage I-II, Stage III-IV, T1–2 stage, T3–4 stage (all *P* < 0.01; Fig. 9).

### Associations between TIICs and our established signature for ccRCC

**Fig. 10:**
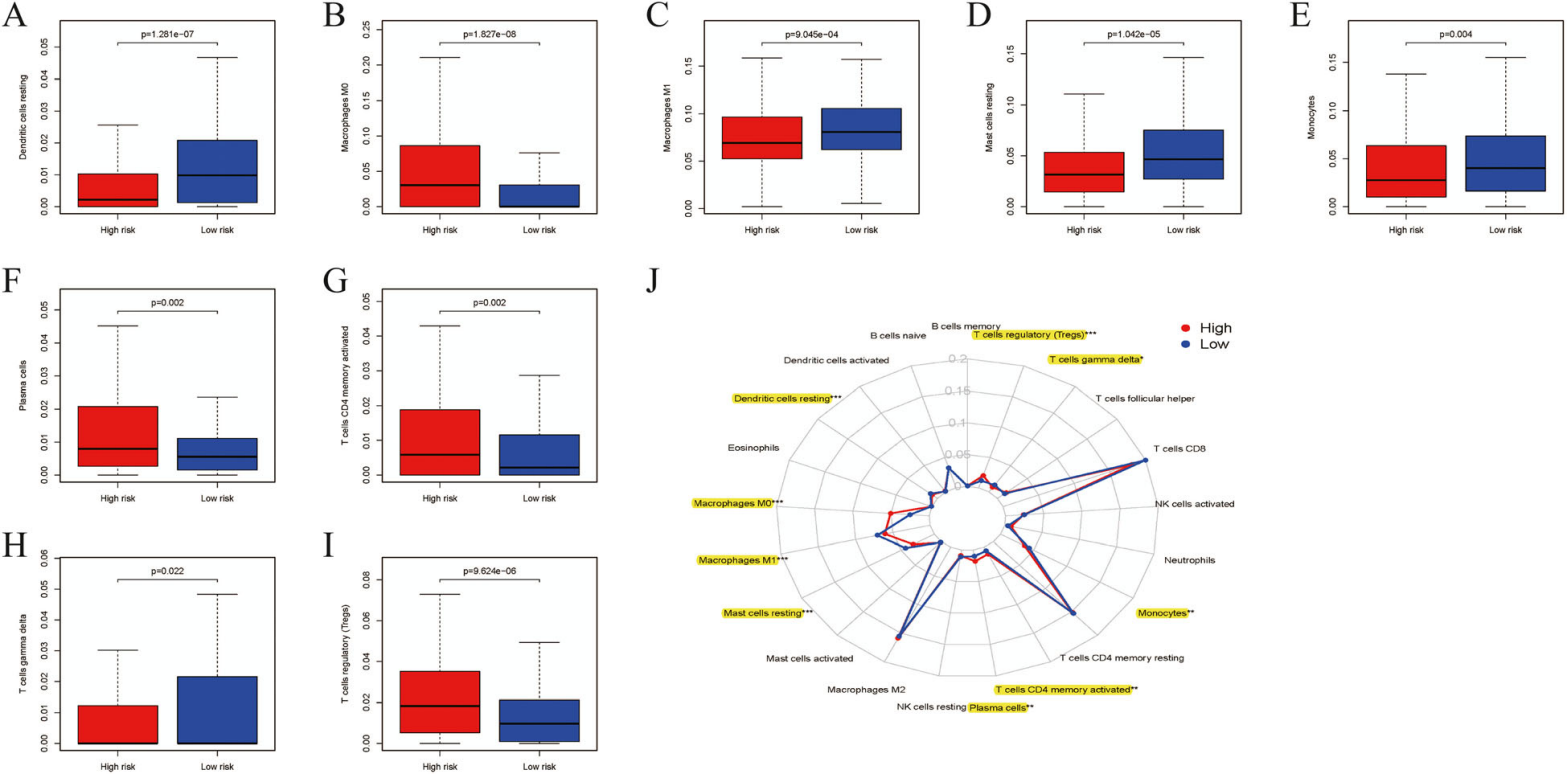
Associations between tumor-infiltrating immune cells (TIICs) and GRGs based riskscore signature in ccRCC; **a** Dendritic cells resting between high- and low-risk ccRCC patients; **b** Macrophages M0 between high- and low-risk ccRCC patients; **c** Macrophages M1 between high- and low-risk ccRCC patients; **d** Mast cells resting between high- and low-risk ccRCC patients; **e** Monocytes between high- and low-risk ccRCC patients; **f** Plasma cells between high- and low-risk ccRCC patients; **g** T cells CD4 memory activated between high- and low-risk ccRCC patients; **h** T cells gamma delta between high- and low-risk ccRCC patients; **i** T cells regulatory (Tregs) between high- and low-risk ccRCC patients; **j** Radar chart showed the difference of immune cell infiltration abundances in ccRCC subtypes

As displayed in Fig. 10a-i**,** nine out of 21 TIICs (T cells CD4 memory activated, Plasma cells, T cells gamma delta, T cells regulatory (Tregs), Macrophages M0, Monocytes, Macrophages M1, Mast cells resting, Dendritic cells resting) were highly associated with high- and low- risk ccRCC patients stratified by our established riskScore (all *P* < 0.05). As detailed in Fig. 10j, radar chart showed the relationships of 21 TIICs between high-risk and low-risk ccRCC patients and nine out of these 21 TIICs were statistically significant (all P < 0.05).

## Discussion

According to GLOBOCAN statistics, there were approximately 403,262 new cases of kidney cancers and 175,098 death worldwide in 2018 [15]. Of all human malignancies, kidney cancer accounted for about 2–3% [2]. At present, few studies had focused on the expression pattern of GRGs and their roles in predicting ccRCC survival. Hence, we identified differentially expressed GRGs between tumor samples and normal tissues based on TCGA raw data to construct a PPI network and to perform GO and KEGG pathway enrichment analysis. In addition, we also conducted univariate COX, LASSO and multivariate COX regression analyses to establish a signature for predicting the ccRCC patients’ prognosis based on 10 prognostic-related GRGs and further explored its associations with immune infiltration. These works may help to identify novel effective biomarkers for ccRCC prognosis.

According to reports, there are metabolic changes in a variety of cancers, and glycolysis is the most common and widely studied metabolic change [16]. It was the first time for us to identify differently expressed GRGs and then screen out 10 prognostic-related GRGs (ANKZF1, CD44, CHST6, HS6ST2, IDUA, KIF20A, NDST3, PLOD2, VCAN, FBP1), based on TCGA, by univariate/multivariate Cox regression and LASSO regression analyses. Some studies have shown that these GRGs play important roles in the tumorigenesis and progress of various tumors, including renal clear cell carcinoma. For example, the expression of HS6ST2 was up-regulated in tumors of gastric cancer, and related to the poor prognosis [17]. Liep J et al. found that overexpression of miR-145-5p and miR-141-3p could inhibit the migration and invasion of RCC cells by influencing the HS6ST2 expression [18]. Regarding KIF20A, it was reported to be overexpressed in various tumors such as bladder cancer, cervical cancer, glioma, lung adenocarcinoma, ovarian clear cell carcinoma, colorectal cancer, liver cancer, prostate cancer, gastric cancer, etc., and often indicates a poor prognosis and poor clinicopathological features [19–28]. Studies had shown that KIF20A might promote the proliferation, invasion and migration of tumor cells by activating the JAK2/STAT3 pathway [23, 26]. Asahara S et al. has also developed cancer vaccine reagent KIF20–66 for the treatment of pancreatic cancer, which has a beneficial therapeutic effect on advanced pancreatic cancer [29]. Epithelial mesenchymal transformation (EMT) is one of the key steps that cause distant metastasis of tumors [30]. FBP1, PLOD2, VCAN, CD44 are all proved to relate to EMT. Studies have shown that PLOD2 is regulated by many factors, such as HIF-1α, TGF-β and microRNA [31]. FBP1 is a rate-limiting enzyme for gluconeogenesis and has recently been considered as a tumor suppressor for various cancers [32]. It is proved that FBP1 interacts with HIF to inhibit the function of nuclear HIF, inhibit glycolysis and pentose phosphate pathway and inhibit the proliferation of renal cancer cells [10]. FBP1 overexpression inhibits the proliferation, migration, invasion and tumorigenesis of cholangiocarcinoma cells by inhibiting the Wnt/β pathway [33]. FBP1 gene silencing could activate the MAPK pathway and then promote cell EMT, invasion and metastasis in prostate cancer [34]. Several studies have found that PLOD2 induces epithelial-mesenchymal transition (EMT) mainly through the PI3K / AKT signaling pathway [11]. Mitsui Y et al. found that VCAN promotes tumorigenesis and metastasis of ccRCC [35]. VCAN knockdown significantly reduced the proliferation of renal cancer cells and increased apoptosis, which is linked to the changes of several TNF signaling related genes such as TNFα, BID and BAK [35]. TGF-β had the ability to enhance the invasiveness of ovarian cancer cells by up-regulating VCAN in fibroblasts (CAF) [36]. Up-regulated VCAN could also promote the invasion and movement of ovarian cancer cells by up-regulating CD44 and activating the NF-κB signaling pathway, matrix metalloproteinase 9 and hyaluronan-mediated movement receptors [36]. Wu et al. have shown that CD44 + bladder cancer cells have a higher invasive ability [13].

Current research shows that clinical and pathological features such as age and metastasis are not sufficient to accurately evaluate cancer patients’ survival. At present, we are looking for effective biomarkers to predict the survival of cancer patients. However, single gene expression levels will be affected by many factors, making these markers unreliable to be independent prognostic indicators. Therefore, a statistical model composed of multiple related genes is much more accurate in evaluating the prognosis of tumor patients than using a single biomarker, so this model has been extensively used. Few studies have concentrated on the role of glycolysis in the prediction of ccRCC prognosis. We established a new prognostic signature based on the expression of 10 GRGs. Based on this risk scoring model, ccRCC patients were classified into a high-group and low-risk group. Patients with high-risk scores had significantly lower OS compared to those with low-risk scores. In addition, we combined the established riskscore and multiple clinical parameters to construct a nomogram to predict 1-, 3-, and 5-year OS in ccRCC patients. The calibration chart based on the TCGA database shows that the predicted value and the observed value are very close, indicating that the prediction performance of nomogram is very good. Similarly, it is checked in the external verification set and the two internal verification sets. Therefore, our new prognosis nomogram may be better than the original clinical factors to help clinicians predict the survival status for ccRCC and provide specific individualized treatment.

As far as we knew, this was the first signature to predict the OS of ccRCC patients based on GRGs. We successfully established a risk signature based on GRGs and verified it in three verification sets including one external verification data set (ArrayExpress) and two internal verification data sets (test 1 and test 2). Our results remained stable both internally and externally. Of course, this study also had several limitations. Firstly, as a bioinformatics analysis article, our research is retrospective and the clinical information downloaded from the online database was incomplete and limited. Secondly, 10 glycolysis-related genes had not been experimentally verified and our constructed nomogram had not been validated by our own data. Thirdly, due to limited clinical information, we had difficulties in comparing our established signature with other available tools in the clinic, such as the Heng score (IMDC).

## Conclusions

Taken together, 10 GRGs including ANKZF1, CD44, CHST6, HS6ST2, IDUA, KIF20A, NDST3, PLOD2, VCAN, FBP1 were selected out and utilized to establish a novel signature. The GRGs based signature was successfully established and verified externally and internally for predicting OS, helping clinicians better and more intuitively predict ccRCC patients’ survival. As an independent prognostic factor, our established signature showed excellent predictive efficacy for ccRCC and significantly associated with immune infiltration. Further researches were required to verify our findings.

## Supplementary Information

**Additional file 1: Supplement Figure S1.** Workflow chart for identifying the glycolysis signature associated with ccRCC survival.

**Additional file 2: Supplement Figure S2**. Time-dependent ROC analyses for OS prediction by nomogram including1-, 3-, 5-year in both TCGA and ArrayExpress databases. (A-C) 1-, 3-, 5-year in TCGA dataset; (D-F) 1-, 3-, 5-year in ArrayExpress dataset.

**Additional file 3: Supplement Figure S3**. Association between clinicopathologic characteristics (grade, stage, T) and our established riskScore; (A) Distribution of riskscores in grade; (B) Distribution of riskscores in stage; (C) Distribution of riskscores in T stage.

**Additional file 4: Supplementary Figure S4**. Validation of the expression of 10 critical GRGs in ccRCC from HPA database; (A) ANKZF1; (B) CD44; (C) CHST6; (D) FBP1; (E) HS6ST2; (F) IDUA; (G) KIF20A; (H) NDST3; (I) PLOD2; (J) VCAN.

## Data Availability

The RNA-sequencing data of ccRCC samples and normal kidney tissue samples with corresponding clinical information were downloaded from the Cancer Genome Atlas (TCGA) database (https://portal.gdc.cancer.gov/) and the ArrayExpress database (E-MTAB-1980, https://www.ebi.ac.uk/arrayexpress/).

## Abbreviations

GRGs: Glycolysis-related genes
ccRCC: Clear cell renal cell carcinoma
TCGA: The Cancer Genome Atlas
OS: Overall survival
FDR: False discovery rate
FC: Fold change
GO: Gene ontology
KEGG: Kyoto encyclopedia of genes and genome
BP: Biological processes
MF: Molecular functions
CC: Cellular components
PPI: Protein-protein interaction
LASSO: Least absolute contraction and selection operator
HPA: Human Protein Atlas
TIICs: Tumor-infiltrating immune cells
