# Supplementary figures and images for: A novel 10 glycolysis-related genes signature could predict overall survival for clear cell renal cell carcinoma
Source: BMC Cancer. 2021 Apr 9;21:381. doi: 10.1186/s12885-021-08111-0 (PMC8034085; doi:10.1186/s12885-021-08111-0)

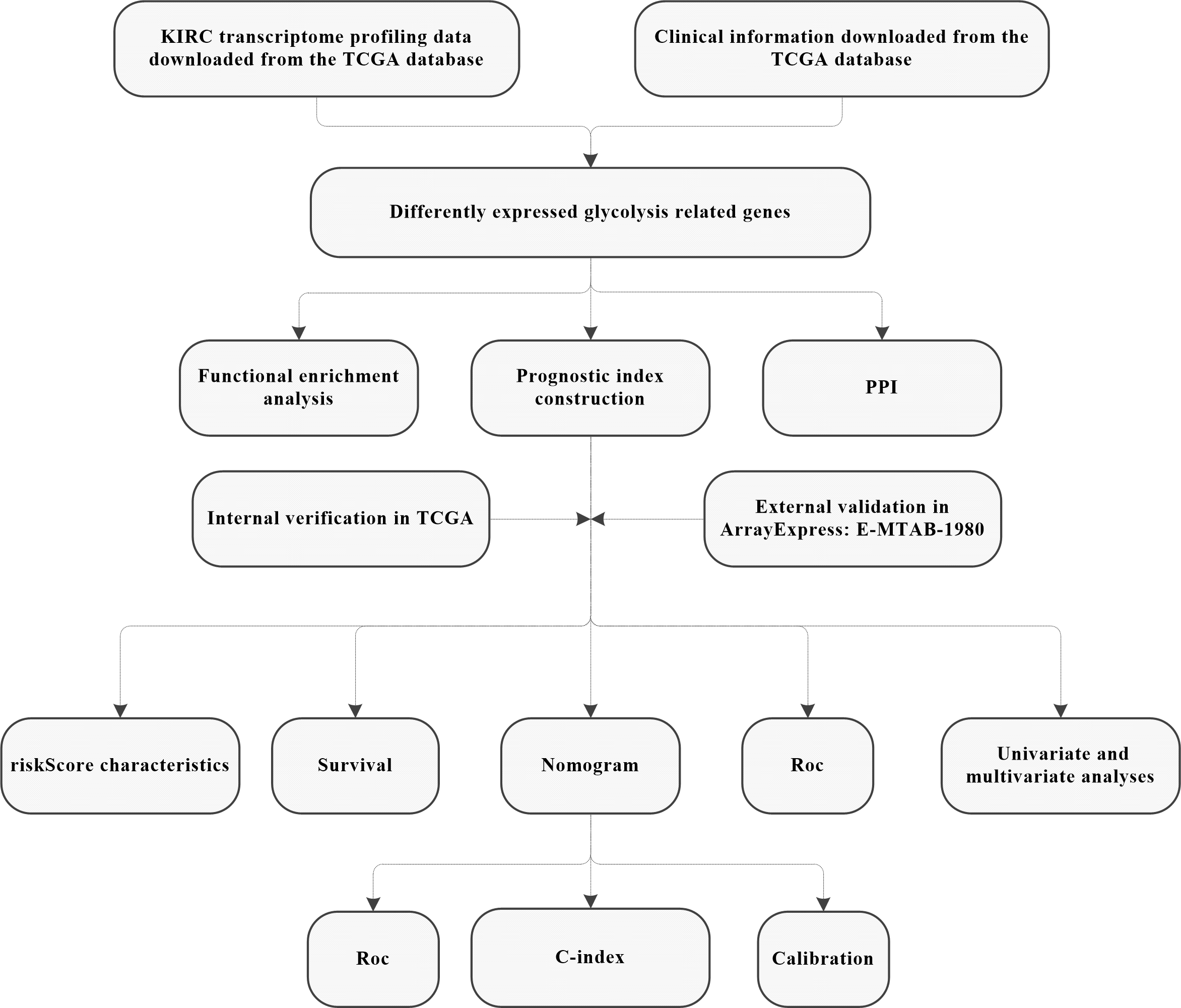

Supplement: Supplementary file 1 — Additional file 1: Supplement Figure S1. Workflow chart for identifying the glycolysis signature associated with ccRCC survival. [file 12885_2021_8111_MOESM1_ESM.tif]

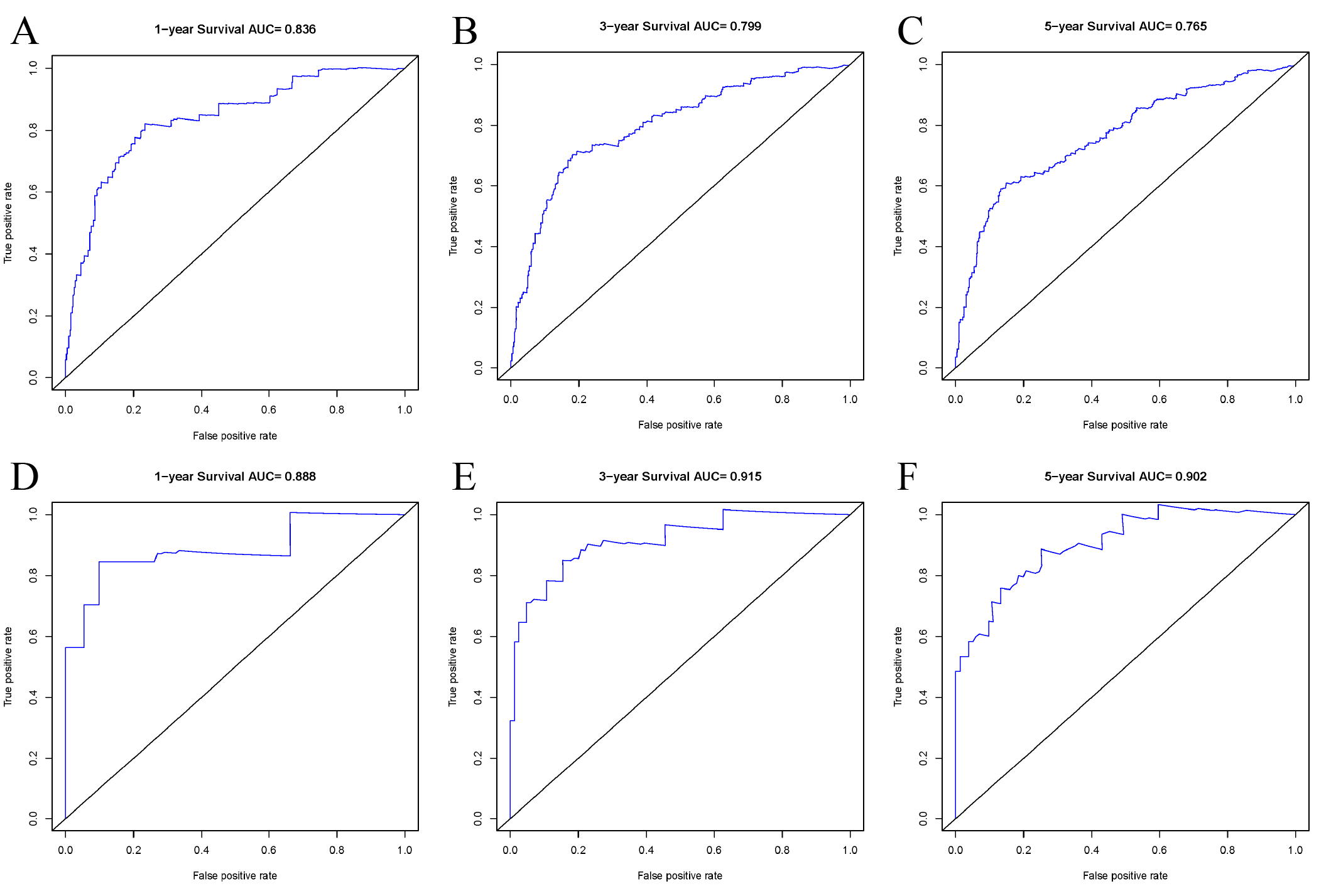

Supplement: Supplementary file 2 — Additional file 2: Supplement Figure S2. Time-dependent ROC analyses for OS prediction by nomogram including1-, 3-, 5-year in both TCGA and ArrayExpress databases. (A-C) 1-, 3-, 5-year in TCGA dataset; (D-F) 1-, 3-, 5-year in ArrayExpress dataset. [file 12885_2021_8111_MOESM2_ESM.tif]

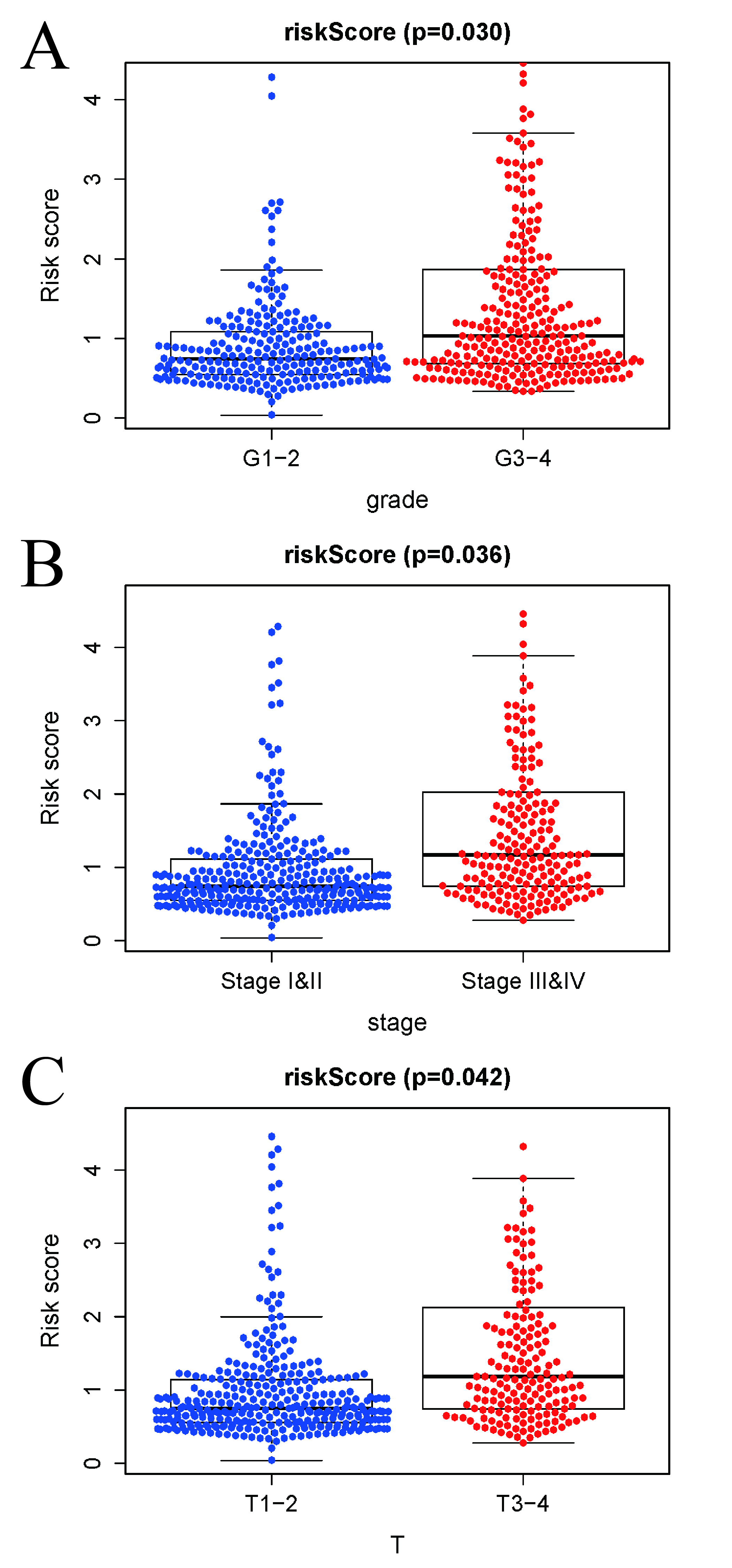

Supplement: Supplementary file 3 — Additional file 3: Supplement Figure S3. Association between clinicopathologic characteristics (grade, stage, T) and our established riskScore; (A) Distribution of riskscores in grade; (B) Distribution of riskscores in stage; (C) Distribution of riskscores in T stage. [file 12885_2021_8111_MOESM3_ESM.tif]

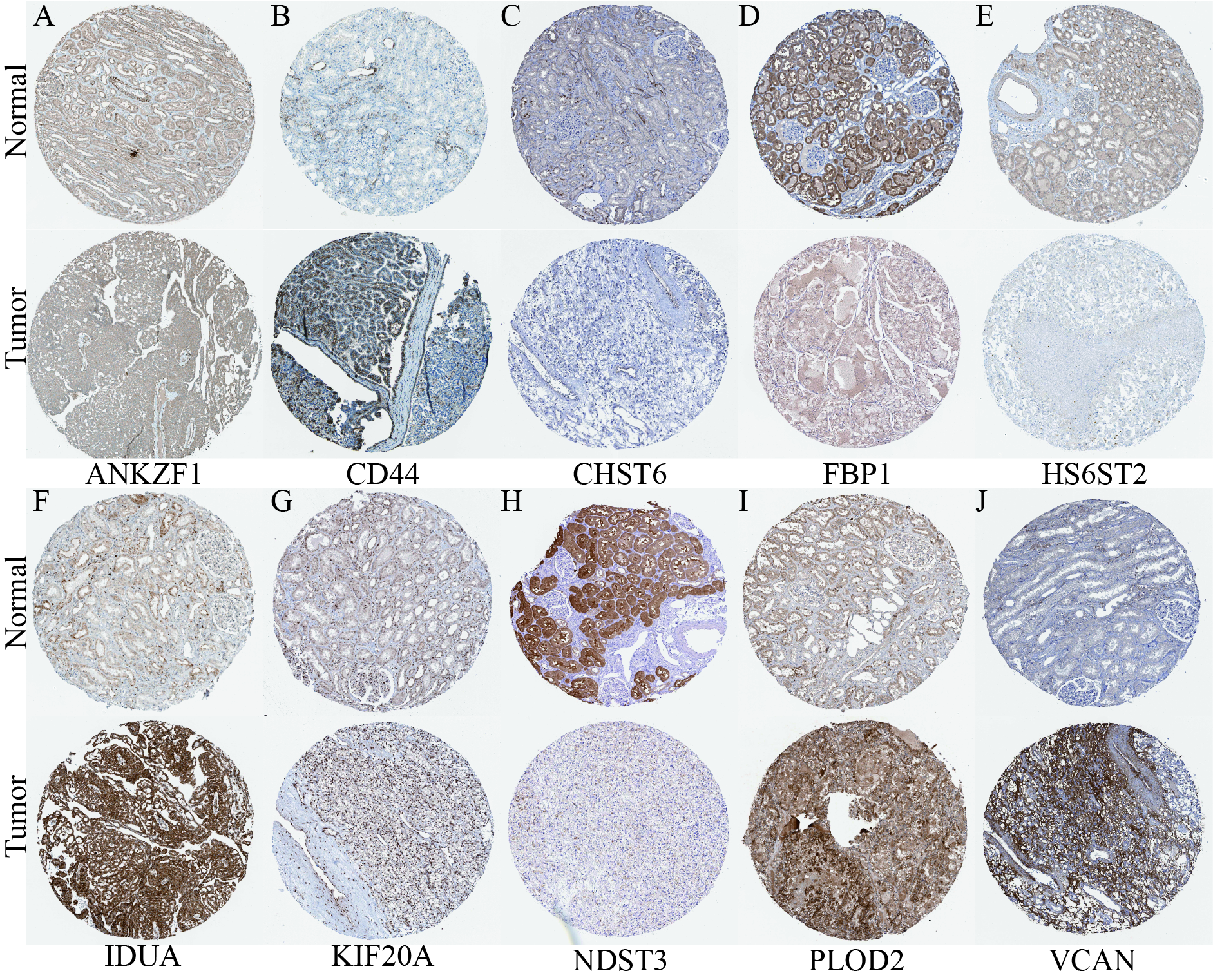

Supplement: Supplementary file 4 — Additional file 4: Supplementary Figure S4. Validation of the expression of 10 critical GRGs in ccRCC from HPA database; (A) ANKZF1; (B) CD44; (C) CHST6; (D) FBP1; (E) HS6ST2; (F) IDUA; (G) KIF20A; (H) NDST3; (I) PLOD2; (J) VCAN. [file 12885_2021_8111_MOESM4_ESM.tif]
